# High prevalence of hypertension and of risk factors for non-communicable diseases (NCDs): a population based cross-sectional survey of NCDS and HIV infection in Northwestern Tanzania and Southern Uganda

**DOI:** 10.1186/s12916-015-0357-9

**Published:** 2015-05-29

**Authors:** Bazil Kavishe, Samuel Biraro, Kathy Baisley, Fiona Vanobberghen, Saidi Kapiga, Paula Munderi, Liam Smeeth, Robert Peck, Janneth Mghamba, Gerald Mutungi, Eric Ikoona, Jonathan Levin, Maria Assumpció Bou Monclús, David Katende, Edmund Kisanga, Richard Hayes, Heiner Grosskurth

**Affiliations:** Mwanza Intervention Trials Unit, National Institute for Medical Research, PO 11936 Mwanza, Tanzania; MRC/UVRI Uganda Research Unit on AIDS / Uganda Virus Research Institute, Entebbe, Uganda; MRC Tropical Epidemiology Group, London School of Hygiene & Tropical Medicine, Keppel Street, London, WC1E 7HT UK; Weill Bugando School of Medicine, Mwanza, Tanzania; Weill Cornell Medical College, New York, USA; Ministry of Health and Social Welfare, Dar es Salaam, Tanzania; Ministry of Health, Kampala, Uganda; School of Public Health, Faculty of Health Sciences, University of the Witwatersrand, Johannesburg, South Africa

**Keywords:** Non-communicable diseases, hypertension, diabetes mellitus, heart failure, obstructive pulmonary disease, HIV infection, NCD risk factors, WHO STEPS survey, Africa

## Abstract

**Background:**

The burden of non-communicable diseases (NCDs) is increasing in sub-Saharan Africa, but data available for intervention planning are inadequate. We determined the prevalence of selected NCDs and HIV infection, and NCD risk factors in northwestern Tanzania and southern Uganda.

**Methods:**

A population-based cross-sectional survey was conducted, enrolling households using multistage sampling with five strata per country (one municipality, two towns, two rural areas). Consenting adults (≥18 years) were interviewed using the WHO STEPS survey instrument, examined, and tested for HIV and diabetes mellitus (DM). Adjusting for survey design, we estimated population prevalences of hypertension, DM, obstructive pulmonary disease, cardiac failure, epilepsy and HIV, and investigated factors associated with hypertension using logistic regression.

**Results:**

Across strata, hypertension prevalence ranged from 16 % (95 % confidence interval (CI): 12 % to 22 %) to 17 % (CI: 14 % to 22 %) in Tanzania, and from 19 % (CI: 14 % to 26 %) to 26 % (CI: 23 % to 30 %) in Uganda. It was high in both urban and rural areas, affecting many young participants. The prevalence of DM (1 % to 4 %) and other NCDs was generally low. HIV prevalence ranged from 6 % to 10 % in Tanzania, and 6 % to 12 % in Uganda. Current smoking was reported by 12 % to 23 % of men in different strata, and 1 % to 3 % of women. Problem drinking (defined by Alcohol Use Disorder Identification Test criteria) affected 6 % to 15 % men and 1 % to 6 % women. Up to 46 % of participants were overweight, affecting women more than men and urban more than rural areas. Most patients with hypertension and other NCDs were unaware of their condition, and hypertension in treated patients was mostly uncontrolled. Hypertension was associated with older age, male sex, being divorced/widowed, lower education, higher BMI and, inversely, with smoking.

**Conclusions:**

The high prevalence of NCD risk factors and unrecognized and untreated hypertension represent major problems. The low prevalence of DM and other preventable NCDs provides an opportunity for prevention. HIV prevalence was in line with national data. In Tanzania, Uganda and probably elsewhere in Africa, major efforts are needed to strengthen health services for the PREVENTION, early detection and treatment of chronic diseases.

**Electronic supplementary material:**

The online version of this article (doi:10.1186/s12916-015-0357-9) contains supplementary material, which is available to authorized users.

## Background

Historically, acute illnesses have been the most important health problems in sub-Saharan Africa (SSA). However, there is growing evidence that the burden of chronic diseases (CDs), in particular that of non-communicable diseases (NCDs), is increasing rapidly in this region [1]. It has been anticipated that NCDs may account for 46 % of deaths in SSA by 2030, compared to 28 % in 2008 [1]. Hypertension, diabetes mellitus (DM), ischemic heart disease and heart failure are of particular concern; however, precise epidemiological data are rare [2–5]. Data on the prevalence of chronic respiratory diseases, such as asthma and chronic obstructive pulmonary diseases (COPD), are even more limited. These diseases are likely to become more prevalent as risk factors become more common [6]. In addition, owing to longer survival following increased access to care and antiretroviral therapy, HIV has also become a CD [7].

Urbanization and associated lifestyle changes, as well as improvements in life expectancy, may explain the increase in NCDs in SSA. More people are engaged in sedentary work and physical activity during leisure time is uncommon [8, 9]. Moreover, populations are increasingly exposed to diets that are high in calories, salt and fat with low fiber content [10]. Fetal and childhood malnutrition, prevalent in Africa, may also contribute to the increasing prevalence of cardiovascular diseases and DM [11, 12]. Various factors including increases in air pollution, the use of biomass fuel and tobacco are likely to result in chronic lung disease [6, 13].

However, data on the burden of NCDs in SSA are limited, and only a few representative community-based studies have been conducted [14]. Data from well-designed epidemiological studies are needed to accurately estimate the prevalence of NCDs in SSA and to facilitate the planning of effective interventions. For this study, we hypothesized that the burden of NCDs in Tanzania and Uganda is high and is associated with modifiable risk factors. We report the prevalence of risk factors for NCDs, the prevalence of selected CDs, and of factors associated with hypertension in northwestern Tanzania and southern Uganda. HIV infection was included for comparison as our work forms part of an ongoing research program that aims to investigate the burden of selected CDs in the general population and within health facilities, and to contribute to the design of intervention programs for the improvement of CD disease services in these countries.

## Methods

### Study design, setting and sampling

We conducted a cross-sectional population survey among adults (≥18-years old) between May 2012 and April 2013. We used stratified, multistage sampling, with five strata in each country: a municipal area (Mwanza city in Tanzania; Entebbe town in Uganda), two district towns (Geita and Kahama in Tanzania; Wakiso and Mpigi in Uganda), and the rural districts corresponding to each district town. We took an independent two-stage self-weighting sample from each stratum, firstly sampling the lowest administrative local authority areas with probability proportional to the number of households, and secondly randomly sampling households within these areas (see Additional file 1 for further details). Households were eligible if they were located within 5 km of a health facility which in both countries is the case for all urban and the great majority of rural homes.

Selected households were visited, verbal consent from household heads obtained, a list of adult household members prepared, and all resident adults invited to participate. Consenting participants were recruited. No replacements were made for households that refused participation or for participants who could not be contacted after three repeat visits.

### Sample size

We aimed to measure the prevalence of important conditions with a precision that would provide sufficiently reliable information for intervention planning. For example, we aimed at estimating the prevalence of a condition that occurs in 5 % of the study population with a precision of +/− 3.3 %. Based on pilot work within the project area, we expected an average number of eligible persons per household of 1.76. Using this number, and assuming a design effect of 3, an overall sample size was required of 792 per country. This number of individuals could be expected to be found in 450 households. To allow for possible errors in the assumed number of participants per household and for a lack of response, we increased the sample size by 20 % overall and thus aimed for 540 households with a total of 950 participants per country.

### Data collection

Participants were interviewed in their homes or at a nearby communal location using a structured questionnaire adapted from the World Health Organization (WHO) STEPwise approach to CD risk factor surveillance (STEPS) instrument [15]. We collected information on socio-demographic characteristics, risk factors for NCDs, symptoms related to NCDs, and disease and treatment history. Information about alcohol use was collected using the Alcohol Use Disorders Identification Test (AUDIT) [16].

Physical examinations were conducted to determine weight, height, waist circumference, blood pressure (BP) and lung function. Weight was measured using a digital seca® 813 scale, height using a seca® 213 stadiometer and waist circumference using a 203 cm seca® measuring tape (all seca GmbH & Co. KG., Hamburg, Germany), with the mean of two waist circumference measurements used for analysis. BP was measured with participants seated after resting for at least 15 minutes, using the Omron digital automatic blood pressure monitor model M6 (Omron Health Care Manufacturing Vietnam Co., Ltd, Binh Duong Province, Vietnam) with an inflatable cuff (small, medium or large size depending on the upper arm circumference). We measured BP once on each arm, and then obtained a third measurement from the arm with the highest value. This third measurement was used for analysis. Lung function tests were performed using Vitalograph® micro model 6300 (Vitalograph, Enis, Ireland) and forced expiratory volume in the first second (FEV1) and forced vital capacity (FVC) were recorded. For each participant, we aimed for five satisfactory spirometer tests. The mean of the two highest spirometer results was used for analysis [17].

Blood samples were collected for HIV rapid testing and random blood glucose (RBG). Participants with a RBG result of ≥7 mmol/L were re-visited for a fasting blood glucose (FBG) test within five days of RBG testing.

### Laboratory tests

Whole venous blood was tested for RBG using a portable-battery driven Accu-Check® Aviva (Roche Diagnostics GmbH, Mannheim, Germany) and FBG using HemoCue® Glucose 201 RT (HemoCue AB, Ängelholm, Sweden). HIV testing was performed using approved testing algorithms in each country. In both countries, Determine™ HIV1/2 (Alere Medical Co. Ltd., Mitsudo-shi, Chiba, Japan) was used as a first-line test and negative results were recorded as such. Positive samples were confirmed by Uni-Gold™ HIV (Trinity Biotech, Plc, Bray, Co.Wicklow, Ireland) in Tanzania and HIV 1/2 STAT-PAK® (Chembio Diagnostic Systems Inc, Medford, NY, USA) in Uganda. In case of discrepant results, HIV 1/2 STAT-PAK® in Tanzania and Uni-Gold™ HIV in Uganda were used as tiebreakers.

### Diagnosis of NCDs

Hypertension was defined according to the seventh report of the internationally recognized Joint National Committee as systolic BP ≥140 mmHg and/or diastolic BP ≥90 mmHg, or currently taking medication for hypertension [18]. We further classified hypertension as stage II (systolic BP ≥160 mmHg and/or diastolic BP ≥100 mmHg) or stage I (those with hypertension but not meeting the definition of stage II), among those not on treatment.

DM was defined as RBG >11.1 mmol/L or FBG ≥7 mmol/L or being on diabetes medication. Heart failure was diagnosed, according to the Framingham criteria [19], if the following conditions were present: orthopnea or paroxysmal nocturnal dyspnea (PND), and at least two of three additional heart failure symptoms (edema, reported breathlessness on exertion, heart rate >120 beats per minute); or orthopnea and PND, and at least one of the three additional symptoms. Obstructive lung disease was defined as FEV1/FVC ≤0.7 according to the Global Initiative for Chronic Obstructive Lung Disease [17]. Epilepsy was diagnosed if participants reported to be on antiepileptic treatment or reported that they experienced seizures during the past 12 months.

### Anthropometric classification

Body mass index (BMI, kg/m^2^) was classified as underweight (<18.5), normal (18.5 to < 25), overweight (25 to <30) and obese (≥30). Waist circumference >94 cm and >80 cm was classified as above normal (central obesity) for males and females, respectively [20].

### Statistical analysis

In Uganda, data were entered on Ultra Mobile Personal Computers in the field, using Microsoft Access. In Tanzania, data were collected on paper-based forms in the field, and subsequently double-entered in OpenClinica® version 3.0.1 (OpenClinica, Waltham, MA, USA). Analyses were conducted with Stata Version 13. We used the Stata survey procedures to account for the complex sampling design, and sampling weights to account for differential probability of selection between strata and, in Tanzania, between clusters (see Additional file 1).

We tabulated the population socio-demographic characteristics, and prevalences of CDs and potential NCD risk factors, stratified by country and location (municipalities, district towns, and rural), and by sex for the prevalences of potential NCD risk factors and hypertension. In addition, the weighted estimates of CD prevalence in each location were age-standardized using the WHO world population aged 18+ as reference [21].

We investigated factors associated with hypertension, combining data from both countries and using logistic regression to estimate odds ratios (OR) and 95 % confidence intervals (CI). We did not investigate risk factors for other NCDs because the prevalences were low. We used the Stata survey procedures to adjust the standard errors for the survey design. We adjusted for age, sex and stratum *a priori* in all models, so comparisons were essentially within the (approximately self-weighted) strata and sampling weights were not applied. Potential determinants of hypertension were examined using a conceptual framework with three levels [22]. Socio-demographic factors were added to the stratum, age and sex-adjusted analysis and retained if associated with hypertension at *P* <0.10. Behavioral factors were then added one by one and retained if they remained associated at *P* <0.10. Associations with anthropometric factors were subsequently determined in a similar way. This strategy allowed us to assess the effects of variables at each level of the framework, adjusted for more distal variables. We estimated the population attributable fraction (PAF) of hypertension for overweight and obese BMI, and central obesity, using the adjusted ORs from the final model. Lastly, we did a similar analysis to explore factors associated with untreated stage II hypertension.

### Ethical considerations

This study was approved by the ethics committees of the Tanzanian National Institute for Medical Research, Uganda Virus Research Institute, Ugandan National Council for Science and Technology, and London School of Hygiene and Tropical Medicine. We obtained written informed consent (witnessed for illiterate participants) from all participants before administering study procedures. Participants were interviewed in privacy to ensure confidentiality, and no personal identifiers were included on the questionnaire. A trained clinician/nurse offered pre-test and post-test counselling for all CDs investigated in this study. Minor ailments were treated on the spot. Participants with a known or newly-diagnosed CD (including HIV infection) were referred to a health center or hospital for further assessment, counselling, and long-term care and treatment. HIV infected individuals were assessed for eligibility to antiretroviral therapy (ART) by existing ART providers or in case of shortage of CD4 tests by the research teams.

## Results

### Population characteristics

**Fig. 1:**
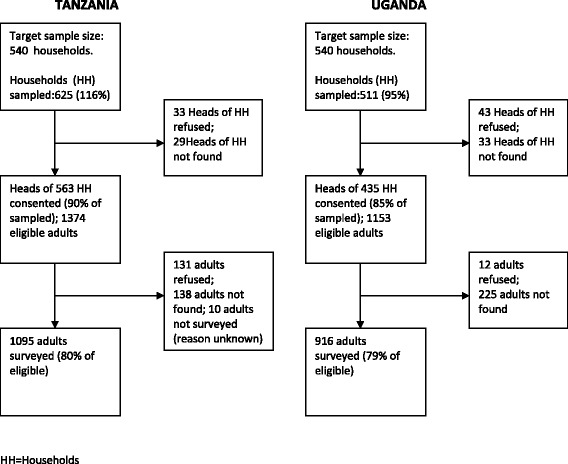
Households and participants sampled and reasons for non-participation

In Tanzania, we enrolled 175 adults in Mwanza municipality, 344 in district towns and 576 in rural communities, providing a total of 1,095 participants from 563 households (Fig. 1). In Uganda, we enrolled 206 people in Entebbe municipality, 278 in district towns and 432 in rural communities, resulting in a total of 916 participants from 435 households. Assuming that the numbers of people within households that did not participate in the survey were similar to those that did, we estimate that we enrolled about 72 % of the targeted study population in Tanzania and 68 % in Uganda. The median age of the study sample was highest in rural areas in both Tanzania and Uganda (33 years, interquartile range (IQR) 24 to 49 and 35 years, IQR 24 to 49, respectively; Table 1). Women made up a larger proportion of the study sample in all strata in both countries (52 % to 62 %), and this difference was largest in Entebbe town.

**Table 1:** Characteristics of study population

Tanzania - Total respondents	Mwanza municipality (number = 175)		District towns (number = 344)		Rural (number = 576)	
	Weighted % (95 % CI)^a^	Unweighted N^b^	Weighted % (95 % CI)^a^	Unweighted N^b^	Weighted % (95 % CI)^a^	Unweighted N^b^
Sex						
Male	44.3 % (35.6–53.4)	77	44.6 % (37.9–51.6)	153	48.2 % (44.1–52.3)	275
Female	55.7 % (46.6–64.4)	98	55.4 % (48.4–62.1)	191	51.8 % (47.7–55.9)	301
Age (years)						
18–24	30.2 % (21.4-40.6)	53	31.6 % (28.4-35.1)	110	26.1 % (21.7-31.0)	145
25–34	32.2 % (19.9-47.6)	57	38.4 % (31.2-46.2)	130	26.9 % (22.5-31.9)	156
35–44	15.8 % (9.5 -25.0)	29	15.2 % (11.4-19.9)	52	15.9 % (13.1-19.2)	93
≥45	21.8 % (16.1-29.0)	36	14.8 % (9.8 -21.6)	52	31.1 % (26.8-35.7)	182
Marital status						
Married/living as married	55.6 % (45.5–65.3)	98	60.6 % (54.1–66.7)	205	70.5 % (67.1–73.8)	410
Divorced/separated/widowed	16.4 % (11.8–22.3)	27	12.6 % (7.9–19.6)	46	17.3 % (13.1–22.5)	96
Single	28.0 % (20.6–36.8)	50	26.8 % (21.3–33.2)	93	12.2 % (9.3–15.7)	70
Education						
None/incomplete primary	21.1 % (14.6–29.6)	34	20.5 % (15.3–27.0)	72	49.2 % (41.7–56.7)	276
Completed primary	48.5 % (43.7–53.4)	85	49.9 % (44.6–55.2)	170	42.9 % (37.4–48.6)	254
Secondary	27.0 % (20.5–34.7)	51	24.7 % (18.4–32.2)	85	7.5 % (5.0–11.3)	44
Above secondary	3.4 % (1.2–9.0 )	5	4.9 % (2.7–8.6 )	17	0.4 % (0.1–1.5 )	2
Monthly income (USD)^c^						
Weighted median (IQR)	38 USD (19–114)		57 USD (25–127)		19 USD (10–38)	
Uganda - Total respondents	Entebbe municipality (number = 206)		District towns (number = 278)		Rural (number = 432)	
Sex						
Male	38.3 % (33.6–43.3)	79	41.0 % (34.4–48.1)	108	42.5 % (37.3–47.9)	188
Female	61.7 % (56.7–66.4)	127	59.0 % (51.9–65.6)	170	57.5 % (52.1–62.7)	244
Age (years)						
18–24	30.1 % (23.1-38.1)	62	37.6 % (29.4-46.6)	94	25.8 % (20.3-32.2)	105
25–34	35.4 % (29.4-41.9)	73	32.2 % (25.9-39.1)	87	22.4 % (17.5-28.3)	95
35–44	15.5 % (12.3-19.4)	32	11.5 % (7.0 -18.4)	45	17.6 % (14.4-21.2)	78
≥45	18.9 % (16.5-21.7)	39	18.7 % (15.2-22.8)	52	34.2 % (27.0-42.3)	154
Marital status						
Married/living as married	44.7 % (34.7–55.0)	92	52.7 % (44.7–60.6)	157	54.8 % (47.0–62.4)	245
Divorced/separated/widowed	17.0 % (11.7–24.1)	35	16.1 % (10.3–24.5)	45	23.0 % (18.7–27.9)	102
Single	38.3 % (29.6–47.9)	79	31.2 % (25.2–37.9)	76	22.2 % (16.3–29.5)	85
Education						
None/incomplete primary	17.5 % (9.7–29.4)	36	22.2 % (18.4–26.4)	67	42.3 % (32.5–52.8)	198
Completed primary	12.6 % (7.4–20.8)	26	11.7 % (6.3–20.6)	34	18.5 % (15.4–22.0)	83
Secondary	56.3 % (49.5–62.9)	116	52.8 % (45.0–60.5)	145	32.1 % (26.3–38.5)	125
Above secondary	13.6 % (8.6–20.9)	28	13.3 % (8.4–20.5)	32	7.1 % (3.8–12.9)	26
Monthly income (USD)^d^						
Weighted median (IQR)	43 USD (0–109)		40 USD (0–121)		20 USD (0–60)	

^a^Weighted estimates, adjusted for survey design with sampling weights applied. See Additional file 1 on sampling methods for more details; ^b^actual number of respondents, without sampling weights applied; ^c^data missing for 3 participants from Mwanza municipality, 20 from district towns and for 39 rural participants; ^d^Missing for 1 participant from Entebbe municipality and 2 rural participants. CI, confidence interval; IQR, interquartile range

In Tanzania, a higher proportion (56 % to 71 %) of study participants were married than in Uganda (45 % to 55 %), and this proportion was higher in rural than urban areas. The level of education varied, with small proportions having received post-secondary education, and 40 % to 50 % of participants from rural areas in both countries not having completed primary education. Participants from Uganda reported completion of secondary education more often than those from Tanzania (32 % to 56 % versus 8 % to 27 %). The median individual monthly monetary income was 38 to 43 US dollars (US$), 40 to 57 USD and 19 to 20 USD in municipal areas, district towns and rural areas, respectively, and was similar across countries except for district towns in Tanzania from where the highest median income was reported (57 USD).

### Prevalence of risk factors for NCDs

The proportion of current smokers among men was substantially higher than among women (12 % to 23 % versus 1 % to 3 %) (Tables 2 and 3), and was consistently higher in Tanzania than in Uganda, while there was no clear trend across countries among women. In both countries, over 60 % of men and over 90 % of women had never smoked. The proportion who reported drinking alcohol during the past 12 months was also higher among men than women (15 % to 29 % versus 4 % to 14 % in Tanzania; 33 % to 58 % versus 29 % to 35 % in Uganda), and was higher in Uganda than in Tanzania for both men and women. The prevalence of problem drinking based on the AUDIT scale ranged across strata from 6 % to 15 % among men in Tanzania and 6 % to 12 % in Uganda; and from 1 % to 6 % among women in Tanzania and from 1 % to 2 % in Uganda.

**Table 2:** Population prevalence of risk factors for NCDs among *men* (≥18 years)

Tanzania – Total men	Mwanza municipality (number = 77)		District towns (number = 153)		Rural (number = 275)	
	Weighted % (95 % CI)^a^	Unweighted N^b^	Weighted % (95 % CI)^a^	Unweighted N^b^	Weighted % (95 % CI)^a^	Unweighted N^b^
Smoking						
Current smoker	18.6 % (7.9–38.0)	14	14.8 % (8.2–25.1)	23	22.7 % (17.1–29.5)	64
Ex–smoker	8.7 % (3.4–20.5)	6	16.3 % (8.6–28.7)	23	15.6 % (11.7–20.4)	45
Never smoked	72.7 % (54.4–85.6)	57	68.9 % (58.9–77.5)	107	61.7 % (54.4–68.5)	166
Alcohol consumption						
Never drinks	58.8 % (51.3–65.9)	46	49.6 % (41.0–58.2)	77	54.8 % (48.2–61.3)	148
No drinking in past 12 months	12.7 % (8.7–18.3)	10	24.2 % (18.0–31.8)	36	30.0 % (25.1–35.3)	86
Drinking in past 12 months	28.5 % (23.1–34.5)	21	26.2 % (18.6–35.6)	40	15.2 % (11.8–19.4)	41
AUDIT scale (among drinkers)^c^						
Non problem drinking	48.4 % (35.6–61.4)	10	49.5 % (30.2–68.9)	19	61.1 % (44.6–75.4)	24
Problem drinking	51.6 % (38.6–64.4)	11	50.5 % (31.1–69.8)	20	38.9 % (24.6–55.4)	17
Eats fewer than one serving of fruit/vegetables per day^d^	28.9 % (20.0–39.7)	17	28.0 % (19.2–39.0)	31	40.1 % (31.5–49.5)	78
Eats fruit/vegetables fewer than 5 days/week	25.3 % (17.9–34.3)	20	24.3 % (17.5–32.8)	38	33.8 % (24.6–44.5)	86
Days of vigorous physical activity/week^e^						
None	29.8 % (19.1–43.1)	25	35.0 % (27.8–42.9)	53	21.3 % (16.9–26.5)	59
1–2	7.0 % (2.6–17.7)	7	7.9 % (4.6–13.2)	12	6.4 % (3.6–11.2)	19
3–4	11.0 % (3.9–27.4)	8	7.5 % (4.3–13.0)	12	8.1 % (5.2–12.3)	23
5+	52.3 % (35.6–68.5)	37	49.6 % (39.0–60.3)	76	64.2 % (59.0–69.0)	174
BMI category (kg/m^2^)^f^						
Underweight (<18.5)	15.5 % (9.6–24.1)	11	7.0 % (3.5–13.7)	10	17.2 % (12.7–23.0)	44
Normal (18.5– < 25)	71.1 % (63.5–77.7)	54	78.3 % (70.8–84.3)	120	77.6 % (72.4–82.1)	214
Overweight (25– < 30)	8.4 % (4.4–15.5)	6	8.0 % (4.7–13.3)	12	4.3 % (2.0–9.0 )	13
Obese (≥30)	5.0 % (2.3–10.4)	4	6.7 % (3.1–13.6)	10	0.8 % (0.2–3.3 )	2
Waist circumference >94 cm^f^	10.6 % (4.4–23.1)	8	13.4 % (8.1–21.4)	20	1.6 % (0.7–3.9 )	5
Uganda– Total men	Entebbe municipality (number = 79)		District towns (number = 108)		Rural (number = 188)	
Smoking^g^						
Current smoker	17.7 % (8.6–33.1)	14	12.2 % (6.3–22.3)	10	17.4 % (11.6–25.4)	33
Ex–smoker	15.2 % (8.9–24.8)	12	11.0 % (6.1–19.0)	12	16.0 % (11.7–21.6)	30
Never smoked	67.1 % (52.8–78.8)	53	76.8 % (63.8–86.2)	85	66.5 % (56.2–75.5)	125
Alcohol consumption						
Never drinks	26.6 % (16.7–39.6)	21	44.8 % (35.7–54.1)	43	28.7 % (21.1–37.8)	52
No drinking in past 12 months	15.2 % (7.6–27.9)	12	22.0 % (12.9–35.0)	25	20.5 % (15.9–26.0)	42
Drinking in past 12 months	58.2 % (44.0–71.2)	46	33.2 % (23.5–44.6)	40	50.8 % (41.6–60.0)	94
AUDIT scale (among drinkers)^h^						
Non problem drinking	80.0 % (70.1–87.2)	36	86.5 % (71.7–94.1)	37	88.1 % (75.2–94.8)	83
Problem drinking	20.0 % (12.8–29.9)	9	13.5 % (5.9–28.3)	3	11.9 % (5.2–24.8)	11
Eats fewer than one serving of fruit/vegetables per day	41.8 % (29.8–54.7)	33	59.9 % (51.9–67.4)	70	70.0 % (61.8–77.0)	135
Eats fruit/vegetables fewer than 5 days/week	39.2 % (28.4–51.2)	31	60.9 % (53.8–67.5)	65	58.0 % (47.9–67.4)	114
Days of vigorous physical activity/week^e^						
None	59.5 % (52.4–66.2)	47	68.8 % (56.0–79.3)	75	72.5 % (63.7–79.8)	140
1–2	12.7 % (4.8–29.2)	10	9.3 % (4.3–18.8)	9	4.2 % (1.6–10.6)	7
3–4	10.1 % (5.6–17.6)	8	0.4 % (0.1–1.1 )	4	6.0 % (3.1–11.5)	11
5+	17.7 % (7.5–36.3)	14	21.5 % (11.8–36.1)	20	17.3 % (11.3–25.5)	30
BMI category (kg/m^2^)^i^						
Underweight (<18.5)	2.6 % (0.7–8.8 )	2	8.1 % (3.3–18.7)	9	16.0 % (10.6–23.4)	30
Normal (18.5– < 25)	85.7 % (75.1–92.3)	66	71.4 % (60.2–80.5)	80	74.8 % (67.6–80.9)	142
Overweight (25– < 30)	9.1 % (3.0–24.3)	7	14.2 % (7.6–25.0)	12	8.5 % (4.8–14.7)	15
Obese (≥30)	2.6 % (0.7–9.0 )	2	6.3 % (3.0–12.8)	5	0.7 % (0.1–4.7 )	1
Waist circumference >94 cm^i^	7.6 % (3.1–17.7)	6	15.6 % (9.2–25.2)	12	3.5 % (1.5–8.0 )	6

^a^Weighted estimates, adjusted for survey design with sampling weights applied. See footnote 1 of Table 1; ^b^actual number of respondents, without sampling weights applied; ^c^missing for 1 participant from district towns. ^d^missing for 14 participants from Mwanza municipality, 42 from district towns and for 82 rural participants (majority did not recall how many servings of vegetables they ate); ^e^defined as spending at least 10 minutes continuously in vigorous–intensity activity per day (as per WHO STEPS Survey questionnaire); ^f^BMI missing for 2 participants from Mwanza municipality, 1 from district towns and for 2 rural participants. Waist circumference data missing for 1 participant from Mwanza municipality; ^g^smoking data missing for 1 participant from Entebbe municipality; ^h^AUDIT score missing for 1 participant from Entebbe municipality; ^i^BMI data missing for 2 participants from Entebbe municipality and 2 from district towns. Waist circumference data missing for 2 participants from district towns. AUDIT, Alcohol Use Disorders Identification Test; BMI, body mass index; CI, confidence interval; NCDs, non-communicable diseases

**Table 3:** Population prevalence of risk factors for NCDs among *women* (≥18 years)

Tanzania– Total women	Mwanza municipality (number = 98)		District towns (number = 191)		Rural (number = 301)	
	Weighted % (95 % CI)^a^	Unweighted N^b^	Weighted % (95 % CI)^a^	Unweighted N^b^	Weighted % (95 % CI)^a^	Unweighted N^b^
Smoking						
Current smoker	2.9 % (0.8–10.0)	3	1.3 % (0.3–6.3 )	3	1.2 % (0.3–5.1 )	3
Ex–smoker	3.5 % (1.2–9.7 )	3	1.1 % (0.3–4.2 )	2	1.3 % (0.5–3.4 )	4
Never smoked	93.6 % (85.4–97.3)	92	97.6 % (93.5–99.1)	186	97.6 % (94.9–98.9)	294
Alcohol consumption						
Never drinks	75.2 % (61.7–85.1)	75	66.4 % (58.6–73.4)	128	76.5 % (68.7–82.8)	231
No drinking in past 12 months	11.2 % (6.2–19.3)	11	24.2 % (18.5–30.9)	44	19.1 % (13.2–26.9)	59
Drinking in past 12 months	13.6 % (6.2–27.1)	12	9.4 % (5.8–14.8)	19	4.4 % (2.5–7.4 )	11
AUDIT scale (among drinkers)^c^						
Non problem drinking	58.6 % (30.8–81.8)	7	78.6 % (59.2–90.3)	14	72.4 % (41.2–90.8)	8
Problem drinking	41.4 % (18.2–69.2)	5	21.4 % (9.7–40.8)	4	27.6 % (9.2–58.8)	3
Eats fewer than one serving of fruit/vegetables per day^d^	27.9 % (16.0–44.0)	26	22.3 % (14.1–33.3)	35	34.8 % (24.7–46.5)	75
Eats fruit/vegetables fewer than 5 days/week	31.0 % (18.8–46.7)	31	19.7 % (12.4–29.9)	40	25.7 % (18.0–35.1)	72
Days of vigorous physical activity/week^e,f^						
None	59.6 % (48.2–70.1)	58	65.7 % (56.8–73.7)	127	34.0 % (27.4–41.2)	103
1–2	4.9 % (1.9–12.1)	5	5.5 % (3.3–9.0 )	10	3.2 % (2.0–5.2 )	10
3–4	5.4 % (2.0–13.8)	5	3.7 % (1.7–7.7 )	7	9.3 % (5.8–14.5)	28
5+	30.1 % (21.2–40.6)	29	25.1 % (17.6–34.5)	47	53.5 % (45.2–61.7)	160
BMI category (kg/m^2^) ^g,h^						
Underweight (<18.5)	10.8 % (7.2–16.0)	11	7.4 % (4.1–13.2)	14	9.8 % (6.7–14.1)	25
Normal (18.5– < 25)	56.1 % (47.4–64.5)	53	53.3 % (45.7–60.7)	98	74.4 % (68.1–79.9)	204
Overweight (25– < 30)	24.0 % (17.9–31.5)	22	17.6 % (13.2–23.1)	32	12.8 % (8.8–18.1)	37
Obese (≥30)	9.0 % (4.9–15.9)	9	21.7 % (14.3–31.6)	38	3.0 % (1.2–7.3 )	8
Waist circumference >80 cm^g^	40.2 % (30.6-50.6)	37	47.5 % (38.6-56.5)	85	32.4 % (26.4-39.1)	93
Uganda– Total women	Entebbe municipality (n = 127)		District towns (n = 170)		Rural (n = 244)	
Smoking						
Current smoker	1.6 % (0.4–5.7 )	2	3.3 % (1.1–9.0 )	5	2.1 % (0.9–4.9 )	6
Ex–smoker	3.9 % (1.5–9.6 )	5	1.1 % (0.2–6.8 )	2	4.1 % (2.3–7.3 )	9
Never smoked	94.5 % (87.3–97.7)	120	95.6 % (90.4–98.1)	163	93.7 % (89.3–96.4)	229
Alcohol consumption						
Never drinks	36.2 % (27.4–46.0)	46	46.1 % (37.0–55.5)	78	45.9 % (38.4–53.6)	115
No drinking in past 12 months	28.3 % (18.3–41.1)	36	23.0 % (16.5–31.0)	38	25.6 % (18.1–34.8)	61
Drinking in past 12 months	35.4 % (25.0–47.5)	45	30.9 % (26.8–35.3)	54	28.5 % (20.6–37.8)	68
AUDIT scale (among drinkers)						
Non problem drinking	97.8 % (83.6–99.7)	44	96.6 % (81.5–99.5)	53	94.5 % (84.2–98.2)	64
Problem drinking	2.2 % (0.3–16.4)	1	3.4 % (0.5–18.5)	1	5.5 % (1.8–15.8)	4
Eats fewer than one serving of fruit/vegetables per day	46.5 % (36.0–57.2)	59	58.1 % (48.6–67.1)	112	63.5 % (54.6–71.5)	158
Eats fruit/vegetables fewer than 5 days/week	44.9 % (35.3–54.8)	57	43.0 % (33.3–53.2)	90	51.2 % (44.0–58.4)	128
Days of vigorous physical activity/week^e^						
None	81.9 % (64.7–91.8)	104	95.6 % (89.4–98.2)	162	91.9 % (84.0–96.1)	225
1–2	7.9 % (3.0–19.3)	10	1.1 % (0.1–8.0 )	2	3.1 % (1.4–6.6 )	7
3–4	2.4 % (0.5–9.6 )	3	2.2 % (0.6–7.5 )	3	0.5 % (0.1–4.0 )	1
5+	7.9 % (3.2–18.1)	10	1.2 % (0.2–8.0 )	3	4.5 % (1.9–9.9 )	11
BMI category (kg/m^2^)^i,j^						
Underweight (<18.5)	1.7 % (0.4–6.2 )	2	0.4 % (0.1–0.9 )	5	10.0 % (6.3–15.5)	26
Normal (18.5– < 25)	55.4 % (42.5–67.5)	67	54.1 % (45.0–63.0)	83	48.2 % (38.7–57.9)	114
Overweight (25– < 30)	24.8 % (17.2–34.4)	30	30.1 % (23.9–37.1)	46	30.3 % (23.6–38.0)	63
Obese (≥30)	18.2 % (9.9–31.0)	22	15.4 % (11.1–21.1)	21	11.4 % (7.4–17.2)	24
Waist circumference >80 cm^k^	39.8 % (26.4–55.0)	49	41.9 % (33.2–51.1)	68	44.6 % (37.9–51.6)	95

^a^Weighted estimates, adjusted for survey design with sampling weights applied. See footnote 1 of Table 1; ^b^actual number of respondents, without sampling weights applied; ^c^missing for 1 participant from district towns; ^d^missing for 10 participants from Mwanza municipality, 39 from district towns and 78 rural participants (majority did not recall how many servings of vegetables they ate); ^e^defined as spending at least 10 minutes continuously in vigorous–intensity activity per day (as per WHO STEPS Survey questionnaire); ^f^missing for 1 participant from Mwanza municipality; ^g^BMI and waist circumference results exclude 35 pregnant women; ^h^missing for 4 rural participants; ^i^BMI and waist circumference results exclude 33 pregnant women; ^j^missing for 3 participants from Entebbe municipality, 1 from district towns and 1 rural participant. ^k^missing for 1 participant from Entebbe municipality. AUDIT, Alcohol Use Disorders Identification Test; BMI, body mass index; CI, confidence interval; NCDs, non-communicable diseases

A substantial proportion of the population reported eating fruit or vegetables on fewer than five days per week: 20 % to 34 % in Tanzania and 39 % to 61 % in Uganda. The low intake of fruit was similar among men and women, and was observed in both urban and rural areas. A large proportion of the population, ranging from 21 % among rural men in Tanzania to 96 % among women from Ugandan district towns, reported no regular vigorous physical activity. This risk factor was more common in Uganda than Tanzania, among women than men in both countries, and in urban areas in Tanzania (but not Uganda). The majority of the population in both countries had a normal BMI, but many were overweight or obese (BMI ≥25 kg/m^2^), ranging from 5 % among rural Tanzanian men to 46 % among women in district towns in Uganda. Women were more affected than men in all strata. Waist circumference measurements generally mirrored these proportions. Despite the high prevalence of overweight, there was also a significant prevalence of malnutrition (BMI <18.5 kg/m^2^) in all areas (0.4 % to 17 %). Malnutrition was more prevalent in rural than urban areas, and in Tanzania than in Uganda, and was similar among men and women (Tables 2 and 3).

### Prevalence of chronic diseases

In both countries and across all strata, hypertension was the most common NCD (Table 4). Hypertension prevalence was highest in rural areas (17 % in Tanzania and 26 % in Uganda), but was only slightly lower in municipal areas and district towns. Overall, only 6 % of individuals with hypertension in each country reported that they were taking medication for their condition, with the lowest proportion in rural Tanzania (3 %). In both countries, even among those on treatment, hypertension was not controlled in the majority. Between 5 % and 9 % of the study population had stage II hypertension.

**Table 4:** Population prevalence of chronic disease (≥18 years) and proportion aware of condition

Tanzania – Total respondents	Mwanza municipality (number = 175)		District towns (number = 344)		Rural (number = 576)	
	Weighted % (95 % CI)^a^	Unweighted N^b^	Weighted % (95 % CI)^a^	Unweighted N^b^	Weighted % (95 % CI)^a^	Unweighted N^b^
HIV positive^c^	9.1 % (5.3–15.3)	15	10.3 % (6.5–15.8)	35	6.4 % (3.7–10.9)	37
Previously diagnosed^d^	6.0 % (0.9–30.0)	1	34.5 % (16.9–57.7)	12	32.4 % (20.4–47.2)	12
On treatment^e^	6.0 % (0.9 -30.0)	1	25.8 % (11.6-48.0)	9	28.7 % (17.1-44.0)	11
Hypertension^f^	16.4 % (11.7–22.4)	29	16.8 % (12.6–21.9)	56	17.6 % (13.9–21.9)	107
Previously diagnosed, on treatment and controlled^d,g,h^	3.7 % (0.6–18.5)	1	1.7 % (0.2–10.9)	1	0	0
Previously diagnosed, on treatment and not controlled^d,g,h^	7.6 % (2.0–24.6)	2	5.4 % (1.5–17.6)	3	2.5 % (0.7–8.7 )	2
Previously diagnosed and not on treatment^d,h^	23.6 % (14.5–35.9)	7	10.4 % (4.7–21.6)	6	4.4 % (1.8–10.6)	4
No previous diagnosis^h^	65.2 % (50.4–77.6)	19	82.5 % (69.0–90.9)	46	93.1 % (82.9–97.4)	101
*Stage I* ^*i*^	*6.0 % (3.5–10.1)*	*11*	*10.7 % (7.4–15.3)*	*35*	*12.1 % (9.6–15.1)*	*74*
*Stage II* ^*i*^	*8.8 % (5.1–14.8)*	*15*	*5.0 % (2.8–8.9 )*	*17*	*5.1 % (3.2–7.9 )*	*31*
Diabetes^j,k^	1.9 % (0.7–5.0 )	3	1.5 % (0.6–3.6 )	5	0.6 % (0.1–2.8 )	3
Previously diagnosed^d^	70.1 % (14.5–97.0)	2	82.4 % (40.3–97.0)	4	37.7 % (16.3–65.2)	1
On treatment^e^	70.1 % (14.5–97.0)	2	41.2 % (7.8 -85.3)	2	37.7 % (16.3-65.2)	1
Heart failure^l^	3.3 % (1.7–6.4 )	6	1.4 % (0.8–2.5 )	5	4.5 % (3.2–6.2 )	22
Previously diagnosed^d^	21.2 % (2.3–75.8)	1	0	0	0	0
COPD/asthma^m,n^	3.5 % (1.5–7.9 )	6	2.7 % (1.3–5.7 )	9	3.9 % (2.2–6.9 )	25
Previously diagnosed^d^	0	0	11.8 % (1.3 -58.0)	1	8.5 % (2.2 -27.3)	2
On treatment^e^	0	1	11.8 % (1.3 -58.0)	1	3.6 % (0.6 -19.8)	1
Epilepsy^o^	0.6 % (0.1–3.8 )	1	0.8 % (0.3–2.2 )	3	1.6 % (0.7–3.4 )	8
Any CD (NCDs and HIV infection)	29.5 % (20.2-40.9)	52	28.0 % (21.7-35.2)	95	29.1 % (25.1-33.4)	172
More than one CD	4.7 % (2.3 -9.4 )	8	4.7 % (2.5 -8.5 )	17	4.1 % (2.3 -7.2 )	25
Any NCD	22.6 % (15.3-32.1)	40	20.8 % (15.3-27.7)	70	25.0 % (21.3-29.1)	148
More than one NCD	2.9 % (1.3 -6.2 )	5	2.0 % (1.0 -4.0 )	7	2.8 % (1.4 -5.4 )	16
Uganda - Total respondents	Entebbe municipality (number = 206)		District towns (number = 278)		Rural (number = 432)	
HIV positive^c^	12.2 % (9.4–15.7)	25	5.5 % (2.4–12.4)	24	11.6 % (8.0–16.6)	49
Previously diagnosed^d^	40.0 % (22.5–60.5)	10	26.4 % (8.0–59.5)	8	51.9 % (34.8–68.5)	25
On treatment^e^	36.0 % (19.5-56.6)	9	26.4 % (8.0 -59.5)	8	49.3 % (33.2-65.6)	24
Hypertension^f^	22.3 % (16.2–30.0)	46	19.2 % (14.0–25.6)	49	26.3 % (23.0–29.9)	111
Previously diagnosed, on treatment and controlled^d,g,h^	2.2 % (0.3–15.4)	1	3.4 % (0.5–20.8)	2	3.4 % (0.9–12.2)	3
Previously diagnosed, on treatment and not controlled^d,g,h^	13.0 % (4.0–34.9)	6	12.8 % (4.3–32.3)	4	5.1 % (2.4–10.5)	5
Previously diagnosed and not on treatment^d,h^	15.2 % (7.4–28.9)	7	6.4 % (1.6–22.2)	2	11.9 % (7.1–19.2)	12
No previous diagnosis^h^	69.6 % (52.0–82.8)	32	77.3 % (59.2–88.9)	41	79.7 % (74.6–84.0)	91
*Stage I* ^*i*^	*12.1 % (7.3–19.3)*	*24*	*11.1 % (7.7–15.9)*	*26*	*16.9 % (14.3–19.9)*	*68*
*Stage II* ^*i*^	*7.5 % (4.6–12.0)*	*15*	*5.4 % (2.8–10.4)*	*17*	*7.8 % (5.4–11.0)*	*35*
Diabetes^j,p^	2.4 % (0.9–6.2 )	5	3.8 % (1.8–7.8 )	7	3.0 % (1.4–6.2 )	10
Previously diagnosed^d^	80.0 % (34.6–96.8)	4	67.0 % (15.4–95.8)	5	40.0 % (22.3–60.8)	4
On treatment^e^	80.0 % (34.6-96.8)	4	50.5 % (11.9-88.5)	4	30.0 % (15.2-50.6)	3
Heart failure^l^	9.2 % (5.5–15.1)	19	2.6 % (1.1–5.9 )	7	3.0 % (1.5–6.0 )	12
Previously diagnosed^d^	5.3 % (0.7–30.9)	1	23.9 % (2.7–78.3)	1	0	0
COPD/asthma^m,q^	3.5 % (1.9–6.3 )	7	2.2 % (0.9–5.1 )	11	9.6 % (6.2–14.4 )	41
Previously diagnosed^d^	0	0	0	0	3.3 % (0.5–18.3 )	1
On treatment^e^	0	0	0	0	3.3 % (0.5–18.3 )	1
Epilepsy^o,r^	0	0	0	0	1.1 % (0.3–3.3 )	5
Any CD (NCDs and HIV infection)	39.3 % (35.4-43.4)	81	28.6 % (22.3-35.8)	84	44.7 % (38.9-50.6)	189
More than one CD	7.3 % (5.5 -9.6 )	15	4.5 % (2.3 -8.4 )	12	8.9 % (6.3 -12.4)	38
Any NCD	31.1 % (26.7-35.8)	64	23.8 % (17.9-30.9)	64	36.7 % (32.8-40.8)	156
More than one NCD	5.3 % (3.5 -8.0 )	11	3.8 % (1.8 -7.8 )	9	5.6 % (3.6 -8.6 )	23

^a^Weighted estimates, adjusted for survey design with sampling weights applied. See footnote 1 of Table 1; ^b^actual number of respondents, without sampling weights applied; ^3^HIV diagnosis missing for 8 participants from Mwanza municipality, 1 from Entebbe municipality, and for 1 rural participant in Uganda (majority refused blood test); ^d^participants reported having been told by a doctor in the past 12 months that they have the condition; denominator is those diagnosed with the condition during the survey; ^e^participants reported currently receiving medication for the condition; denominator is those diagnosed with the condition during the survey; ^f^systolic BP ≥140 and/or diastolic BP ≥90, in 3rd measurement at a single visit, or currently receiving drugs for high blood pressure. Missing for 1 participant from Mwanza municipality and 1 rural participant in Tanzania; ^g^participants reported currently receiving drugs for high blood pressure; ‘controlled’ defined as systolic BP <140 and diastolic BP <90); ^h^denominator is N with hypertension; ^i^stage I: systolic BP ≥140 and <160 and/or diastolic BP ≥90 and <100. Stage II: systolic BP ≥160 and/or diastolic BP ≥100. Excludes participants currently receiving drugs for high blood pressure: 21 in Uganda and 9 Tanzania; ^j^random blood glucose (RBG) >11.1, or RBG 7 to 11.1 and fasting blood glucose (FBG) ≥7 mmol/L, or currently receiving insulin or oral diabetes drugs in the last two weeks; ^k^missing for 3 participants from Mwanza municipality, 7 from district towns and for 12 rural participants (majority refused RBG or did not return for FBG test); ^l^orthopnea or paroxysmal nocturnal dyspnea (PND) and at least 2/3 of edema, breathlessness on excersion (BOE) and heart rate (HR) >120, or orthopnea and PND and at least 1/3 of edema, BOE and HR >120; ^m^FEV1/FVC <0.70; ^n^COPD/asthma diagnosis missing for 4 participants from Mwanza municipality, 8 from district towns and for 17 rural participants (mostly because test was contraindicated or participant unable to perform test); ^o^reports having had a seizure in past 12 months, or currently taking medication for epilepsy; ^p^missing for 1 participant from district towns and for 2 rural participants; ^q^COPD/asthma diagnosis missing for 4 participant from Entebbe municipality, 9 from district towns and for 30 rural participants (majority were unable to perform the spirometry test); ^r^missing for one participant from Entebbe municipality. BP, blood pressure; CD, chronic disease; CI, confidence interval; COPD, chronic obstructive pulmonary disease; NCD, non-communicable disease

Hypertension was more prevalent in older age groups, yet a substantial proportion of people aged under 25-years had hypertension (for example, 6 % to 19 % and 7 % to 17 % of young men in Tanzania and Uganda, respectively), although confidence intervals were wide (Table 5).

**Table 5:** Prevalence of hypertension^a^ by sex and age group

	Municipality (Mwanza /Entebbe)		District towns		Rural	
	Weighted % (95 % CI)^b^	Unweighted N^c^	Weighted % (95 % CI)^b^	Unweighted N^c^	Weighted % (95 % CI)^b^	Unweighted N^c^
Tanzania						
Men						
Age group						
18–24 years	5.7 % (0.9 -29.9)	1 / 22	19.2 % (10.2-33.2)	8 / 42	8.1 % (2.6 -22.7)	5 / 65
25–34 years	5.4 % (1.0 -23.7)	2 / 28	4.2 % (1.1 -14.9)	2 / 55	20.5 % (15.1-27.2)	15 / 70
35–44 years	50.9 % (23.7-77.6)	6 / 11	34.6 % (19.7-53.3)	10 / 30	15.6 % (8.8 -25.9)	8 / 51
≥45 years	35.9 % (18.0-58.7)	6 / 15	37.2 % (20.9-57.0)	9 / 26	25.0 % (16.1-36.5)	26 / 88
Women						
Age group						
18–24 years	3.4 % (0.7 -14.5)	1 / 31	1.8 % (0.2 -11.8)	1 / 68	4.1 % (1.7 -9.8 )	4 / 80
25–34 years	7.6 % (1.9 -25.6)	2 / 29	6.7 % (2.6 -15.9)	5 / 75	9.0 % (4.2 -18.0)	7 / 86
35–44 years	21.2 % (7.9 -45.9)	4 / 18	29.2 % (15.2-48.7)	6 / 22	7.1 % (2.5 -18.8)	4 / 42
≥45 years	36.3 % (20.2-56.2)	7 / 20	58.0 % (40.5-73.7)	15 / 26	41.4 % (27.7-56.6)	38 / 93
Uganda						
Men						
Age group						
18–24 years	7.4 % (1.7 -26.9)	2 / 27	16.9 % (7.6 -33.2)	4 / 35	17.4 % (8.6 -32.0)	7 / 45
25–34 years	26.7 % (12.2-48.7)	8 / 30	14.4 % (6.8 -27.9)	3 / 34	8.6 % (3.0 -22.4)	4 / 45
35–44 years	44.4 % (18.9-73.3)	4 / 9	41.2 % (15.1-73.3)	6 / 19	21.1 % (9.3 -41.1)	7 / 31
≥45 years	76.9 % (58.6-88.7)	10 / 13	70.4 % (43.1-88.3)	12 / 20	44.9 % (33.5-56.7)	31 / 67
Women						
Age group						
18–24 years	2.9 % (0.4 -16.2)	1 / 35	2.8 % (0.5 -15.9)	2 / 59	6.0 % (2.5 -13.8)	3 / 60
25–34 years	7.0 % (2.0 -21.8)	3 / 43	9.9 % (3.6 -24.5)	5 / 53	16.2 % (7.1 -32.8)	7 / 50
35–44 years	26.1 % (10.8-50.6)	6 / 23	1.1 % (0.3 -4.3 )	2 / 26	25.6 % (14.5-41.0)	10 / 47
≥45 years	46.2 % (33.1-59.8)	12 / 26	40.8 % (19.4-66.4)	15 / 32	50.5 % (37.8-63.1)	42 / 87

^a^Systolic BP ≥140 and/or diastolic BP ≥90, in 3rd measurement at a single visit, or currently receiving drugs for high blood pressure; ^b^weighted estimates, adjusted for survey design with sampling weights applied. See footnote 1 of Table 1; ^c^actual number of respondents, without sampling weights applied. BP, blood pressure; CI, confidence interval

The prevalence of DM was around 1 % to 2 % across all strata in Tanzania, and slightly higher in Uganda (2 % to 4 %) (Table 4). Of the 33 participants with DM, 27/33 (82 %) were diagnosed based on elevated RBG and FBG levels, one had high RBG (with no FBG measurement) and five (15 %) had normal RBG levels, but reported to be on diabetes medication. Another 11 received medication but were not controlled (69 % of those on treatment). Across strata, between 38 % and 70 % of patients in Tanzania, and between 40 % and 80 % in Uganda were aware of their diabetic condition, with lower levels in rural than urban areas.

Heart failure prevalence ranged from about 1 % in Tanzanian district towns to 9 % in Entebbe town, with no clear trends by area. The prevalence of obstructive lung disease ranged from 3 % in Tanzanian district towns to 10 % in rural Uganda and was highest in rural areas in both countries. Epilepsy was reported by only a few participants, mostly from rural Tanzania (prevalence 1.6 %).

With the exception of DM, the proportion of the population with CDs who were aware of their condition was low, and this lack of awareness was more prevalent in rural areas. For example, 80 % and 93 % of the population with hypertension in rural Uganda and Tanzania, respectively, were not aware of their condition, compared with 65 % and 70 % in Mwanza and Entebbe municipalities, respectively.

**Fig. 2:**
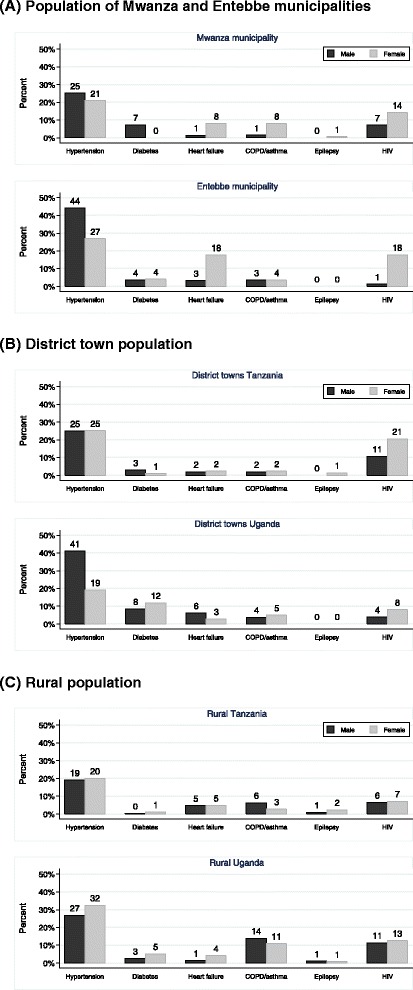
Chronic disease prevalence (age 18+ years), age-standardized to the WHO world population, for 3 study populations (**a**, Municipalities; **b**, District towns; **c** Rural areas)

HIV prevalence for both sexes combined ranged from 6 % (95 % CI 4 % to 11 %) in rural areas to 10 % (CI 6 % to 16 %) in district towns in Tanzania, and from 6 % (CI 2 % to 12 %) in district towns to 12 % (CI 9 % to 16 %) in Entebbe municipality in Uganda (Table 4). It was higher among women than men in all strata, and this difference was particularly strong in municipal areas and district towns in both countries (Fig. 2). Among the HIV positive population, awareness was also low, ranging from 6 % in Mwanza to 35 % in district towns in Tanzania, and from 26 % in district towns to 52 % in rural areas in Uganda.

**Fig. 3:**
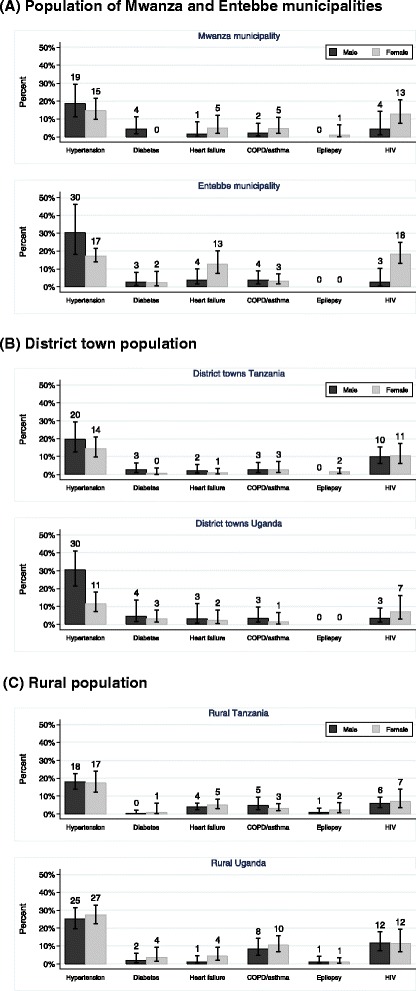
Observed population prevalence of chronic diseases (age 18+ years) with 95 % confidence interval, for 3 study populations (**a**, Municipalities; **b**, District towns; **c** Rural areas)

Age-standardized CD prevalences were generally higher than the observed population prevalences for most conditions (Fig. 2 versus Fig. 3), reflecting the fact that the prevalence of these CDs increases with age, and that the Tanzanian and Ugandan populations are somewhat younger than the average world population. For example, age-standardized hypertension prevalence was 19 % to 25 % among men and 20 % to 25 % among women from Tanzania and 27 % to 44 % among men and 19 % to 32 % among women in Uganda (Fig. 2), while the corresponding observed prevalences were 18 % to 18 % among men and 14 % to 17 % among women from Tanzania, and 25 % to 30 % among men and 11 % to 27 % among women from Uganda (Fig. 3).

Across different strata, between 21 % and 37 % of the survey population had any NCD, and between 2 % and 6 % had more than one NCD (Table 4). This comorbidity occurred more often in Uganda than Tanzania, but there were no systematic differences between strata. The prevalence of diabetes was about four times higher among patients with hypertension than overall, and about two thirds of the patients with diabetes were also hypertensive (data not shown).

### Factors associated with hypertension

Among the socio-demographic variables, age, sex, marital status and education were independently associated with hypertension (Table 6). Hypertension prevalence increased significantly with age: adjusted odds ratio (aOR) = 10.30, 95 % CI 6.41 to 16.54, comparing those ≥45-years old with those <25-years old; decreasing education level (aOR = 1.40, 95 % CI 0.95 to 2.05, comparing those with less than primary to those with secondary or above), and with being divorced, separated or widowed (aOR = 1.49, CI = 1.11 to 2.00, compared with those who were married). Hypertension prevalence was significantly lower among women than men (aOR 0.67, 95 % CI 0.53 to 0.86).

**Table 6:** Factors associated with hypertension^a^

	Number with hypertension / total Number (%)^b^	Age-, sex- and stratum adjusted OR (95 % CI)^c^	Adjusted OR (95 % CI)^c,d^
**Sociodemographic**			
Age group		***P*** **<0.001**	***P*** **<0.001**
18–24 years	39 / 569 (6.9 %)	**1**	**1**
25–34 years	63 / 598 (10.5 %)	**1.61 (1.04 – 2.49)**	**1.62 (1.00 – 2.62)**
35–44 years	73 / 329 (22.2 %)	**4.04 (2.80 – 5.84)**	**4.01 (2.53 – 6.36)**
≥45 years	223 / 513 (43.5 %)	**11.23 (7.84 – 16.10)**	**10.30 (6.41 – 16.54)**
Sex		***P*** **= 0.008**	***P*** **= 0.002**
Male	196 / 878 (22.3 %)	**1**	**1**
Female	202 / 1131 (17.9 %)	**0.72 (0.57 – 0.92)**	**0.67 (0.53 – 0.86)**
Marital status		P = 0.03	***P*** **= 0.02**
Married/living as married	227 / 1205 (18.8 %)	1	**1**
Divorced/separated/widowed	125 / 351 (35.6 %)	1.52 (1.11 -2.07 )	**1.49 (1.11 -2.00 )**
Single	46 / 453 (10.2 %)	1.22 (0.76 -1.96 )	**1.31 (0.81 -2.11 )**
Education		*P* = 0.06	***P*** **= 0.06**
Secondary or above	91 / 675 (13.5 %)	1	**1**
Primary	135 / 651 (20.7 %)	1.62 (1.09 -2.40 )	**1.62 (1.09 -2.40 )**
None/incomplete primary	172 / 683 (25.2 %)	1.45 (0.99 -2.13 )	**1.40 (0.95 -2.05 )**
Monthly income (USD)		*P* = 0.32	*P* = 0.44
Top tertile	119 / 655 (18.2 %)	1	1
Middle tertile	120 / 663 (18.1 %)	1.02 (0.71 -1.46 )	0.96 (0.65 -1.40 )
Lower tertile	159 / 691 (23.0 %)	1.24 (0.90 -1.70 )	1.16 (0.82 -1.64 )
**Behavioral**			**Adjusted OR (95 % CI)** ^**c,e**^
Smoking		*P* = 0.004	***P*** **= 0.001**
Never smoked	320 / 1676 (19.1 %)	1	**1**
Ex–smoker	37 / 153 (24.2 %)	0.59 (0.40 -0.87 )	**0.56 (0.38 -0.82 )**
Current smoker	41 / 179 (22.9 %)	0.57 (0.38 -0.84 )	**0.51 (0.35 -0.77 )**
Alcohol consumption		P = 0.93	*P* = 0.59
Never drinks/no drinking in past 12 months	273 / 1518 (18.0 %)	1	1
Non–problem drinking^7^	100 / 399 (25.1 %)	1.00 (0.74 -1.36 )	1.05 (0.76 -1.43 )
Problem drinking^7^	24 / 89 (27.0 %)	1.11 (0.63 -1.95 )	1.36 (0.75 -2.46 )
Eats fruit/vegetables fewer than five days/week		*P* = 0.69	*P* = 0.60
No	239 / 1238 (19.3 %)	1	1
Yes	159 / 771 (20.6 %)	1.05 (0.81 -1.37 )	1.08 (0.82 -1.42 )
Days of vigorous physical activity/week		*P* = 0.38	*P* = 0.32
None	255 / 1178 (21.6 %)	1.27 (0.89 -1.80 )	1.29 (0.90 -1.85 )
1–4	35 / 221 (15.8 %)	1.08 (0.66 -1.77 )	1.07 (0.65 -1.77 )
5+	108 / 609 (17.7 %)	1	1
**Anthropometric**			**Adjusted OR (95 % CI)** ^**c,f**^
BMI category (kg/m^2^)		*P* <0.001	***P*** **= 0.06**
Underweight (<18.5)	29 / 191 (15.2 %)	0.57 (0.35 -0.93 )	**0.60 (0.37 -0.97 )**
Normal (18.5– < 25)	217 / 1337 (16.2 %)	1	**1**
Overweight (25– < 30)	92 / 309 (29.8 %)	2.00 (1.37 -2.92 )	**1.51 (0.94 -2.43 )**
Obese (≥30)	52 / 155 (33.5 %)	2.15 (1.34 -3.45 )	**1.57 (0.95 -2.59 )**
Waist circumference >94 cm (M)/>80 cm (F)		*P* <0.001	***P*** **= 0.02**
No	235 / 1474 (15.9 %)	1	**1**
Yes	161 / 531 (30.3 %)	2.47 (1.66 -3.69 )	**1.83 (1.12 - 3.01)**

^a^Systolic BP ≥140 and/or diastolic BP ≥90, in 3rd measurement at a single visit, or currently receiving drugs for high blood pressure; ^b^actual number of respondents and proportion with hypertension, without sampling weights applied; ^c^standard errors adjusted for clustering in survey design; ^d^sociodemographic factors adjusted for age, sex, stratum (*a priori*) and all independent sociodemographic predictors of hypertension: marital status and education (variables in bold); ^e^behavioral factors adjusted for age, sex, stratum, marital status, education and independent behavioral predictors of hypertension: smoking (variables in bold); ^f^anthropometric factors adjusted for age, sex, stratum, marital status, education and independent behavioral and anthropometric predictors of hypertension: smoking, BMI category and waist circumference category (variables in bold); ^g^based on AUDIT screening tool. Non-problem drinking defined as AUDIT score <8; problem drinking as AUDIT score ≥8. AUDIT, Alcohol Use Disorders Identification Test; BMI, body mass index; CI, confidence interval; USD, US dollars

Among behavioral risk factors, after adjusting for stratum and socio-demographic risk factors, hypertension prevalence was lower among ex-smokers (aOR 0.56, 95 % CI 0.38 to 0.82) and current smokers (aOR 0.51, 95 % CI 0.35 to 0.77) than never-smokers (*P* <0.001) (Table 6). In the crude analysis (adjusted for sampling stratum only), there was no evidence of an association between smoking and hypertension, but after adjusting for age and sex, the strong inverse association appeared. There was no significant association of hypertension with other behavioral risk factors.

Among anthropometric risk factors, after adjusting for stratum and socio-demographic and behavioral risk factors, both higher BMI and a waist circumference above normal range were associated with hypertension. Participants with a high waist circumference had a two-fold higher odds of hypertension compared with those with normal waist circumference (aOR = 1.83, 95 % CI 1.12 to 3.01).

In an analysis of factors associated with untreated stage II hypertension, we observed generally similar directions of associations including weak evidence of an inverse association with smoking (Table 7). An analysis restricted to patients with hypertension showed that women were two-fold more likely than men to develop stage II hypertension. While men were overall more likely to be hypertensive (Table 6), women were more likely to develop advanced disease (see Supplementary table in Additional file 2).

**Table 7:** Factors associated with untreated stage II hypertension^a^

	Number with stage II hypertension / total Number (%)^b^	Age-, sex- and stratum adjusted OR (95 % CI)^c^	Adjusted OR (95 % CI)^c,d^
**Sociodemographic**			
Age group		***P*** **<0.001**	***P*** **<0.001**
<35 years	17 / 1163 (1.5 %)	**1**	**1**
35–49 years	32 / 448 (7.1 %)	**5.54 (3.18 -9.64 )**	**4.98 (2.90 -8.54 )**
≥50 years	81 / 368 (22.0 %)	**22.20 (13.01-37.89)**	**19.10 (11.48-31.79)**
Sex		***P*** **= 0.31**	***P*** **= 0.41**
Male	50 / 870 (5.7 %)	**1**	**1**
Female	80 / 1109 (7.2 %)	**1.23 (0.82 -1.84 )**	**1.18 (0.79 -1.78 )**
Marital status		*P* = 0.38	*P* = 0.50
Married/living as married	70 / 1191 (5.9 %)	1	1
Divorced/separated/widowed	52 / 336 (15.5 %)	1.34 (0.88 -2.02 )	1.28 (0.85 -1.92 )
Single	8 / 452 (1.8 %)	0.91 (0.43 -1.94 )	1.05 (0.49 -2.23 )
Education		*P* = 0.06	***P*** **= 0.06**
Secondary or above	20 / 664 (3.0 %)	1	**1**
Primary	40 / 644 (6.2 %)	1.96 (1.01 -3.78 )	**1.96 (1.01 -3.78 )**
None/incomplete primary	70 / 671 (10.4 %)	1.91 (1.12 -3.29 )	**1.91 (1.12 -3.29 )**
Monthly income (USD)		*P* = 0.34	*P* = 0.44
Top tertile	15 / 294 (5.1 %)	1	1
Middle tertile	20 / 296 (6.8 %)	1.23 (0.55 -2.74 )	1.14 (0.50 -2.59 )
Lower tertile	32 / 305 (10.5 %)	1.70 (0.81 -3.58 )	1.56 (0.74 -3.26 )
**Behavioral**			**Adjusted OR (95 % CI)** ^e^
Smoking		*P* = 0.20	*P* = 0.14
Never smoked	107 / 1652 (6.5 %)	1	1
Ex–smoker	10 / 150 (6.7 %)	0.56 (0.27 -1.18 )	0.53 (0.25 -1.11 )
Current smoker	13 / 176 (7.4 %)	0.61 (0.25 -1.50 )	0.58 (0.24 -1.41 )
Alcohol consumption		*P* = 0.34	*P* = 0.38
Never drinks/no drinking in past 12 months	87 / 1499 (5.8 %)	1	1
Non-problem drinking^g^	32 / 389 (8.2 %)	1.06 (0.67 -1.69 )	1.08 (0.67 -1.72 )
Problem drinking^g^	10 / 88 (11.4 %)	1.83 (0.81 -4.14 )	1.80 (0.79 -4.12 )
Eats fruit/veg fewer than five days/week		*P* = 0.57	*P* = 0.67
No	76 / 1219 (6.2 %)	1	1
Yes	54 / 760 (7.1 %)	1.15 (0.70 -1.89 )	1.11 (0.68 -1.83 )
Days of vigorous physical activity/week		*P* = 0.31	*P* = 0.31
None	90 / 1155 (7.8 %)	1.29 (0.76 -2.20 )	1.33 (0.78 -2.25 )
1–4	6 / 216 (2.8 %)	0.64 (0.26 -1.56 )	0.66 (0.27 -1.60 )
5+	34 / 607 (5.6 %)	1	1
**Anthropometric**			**Adjusted OR (95 % CI)** ^f^
BMI category (kg/m^2^)		*P* = 0.12	*P* = 0.85
Underweight (<18.5)	13 / 191 (6.8 %)	0.76 (0.33 – 1.72)	0.84 (0.38 -1.87 )
Normal (18.5– < 25)	65 / 1329 (4.9 %)	1	1
Overweight (25– < 30)	29 / 299 (9.7 %)	1.60 (0.88 – 2.91)	1.01 (0.53 -1.93 )
Obese (≥30)	19 / 144 (13.2 %)	2.11 (1.07 – 4.15)	1.25 (0.61 -2.57 )
Waist circumference >94 cm (males)/>80 cm (women)		*P* < 0.001	**P < 0.001**
No	67 / 1468 (4.6 %)	1	**1**
Yes	62 / 507 (12.2 %)	2.58 (1.49 -4.46 )	**2.72 (1.58 -4.67 )**

^a^Systolic BP ≥160 and/or diastolic BP ≥100 in third measurement at a single visit. Excludes those on treatment for hypertension (9 in Tanzania and 21 in Uganda); ^b^actual number of respondents and proportion with hypertension, without sampling weights applied; ^c^standard errors adjusted for clustering in survey design; ^d^sociodemographic factors adjusted for age, sex, stratum (*a priori*) and independent sociodemographic predictors of stage II hypertension: education (variables in bold). ^e^behavioral factors adjusted for age, sex and stratum (*a priori*) and education; ^f^ anthropometric factors adjusted for age, sex, stratum, education and independent behavioral and anthropometric predictors of stage II hypertension: waist circumference category; ^g^based on AUDIT screening tool. Non-problem drinking defined as AUDIT score <8; problem drinking as AUDIT score ≥8. AUDIT, Alcohol Use Disorders Identification Test; BMI, body mass index; CI, confidence interval; USD, US dollars

An analysis of the small group of hypertensive patients who were aware of their condition suggests that older people, women and those with a comparatively higher income were more likely to know their diagnosis. Awareness was more common among ex smokers, but also among those who were less physically active and being overweight.

The adjusted PAF of hypertension due to overweight and obesity was 13 %, and for central obesity (defined by waist circumference above the normal range) was 18 %. The adjusted joint PAF of hypertension for both risk factors was 28 %.

## Discussion

In this population-based survey in northwestern Tanzania and southern Uganda, we observed a high prevalence of hypertension: in different strata the age-standardized prevalence ranged between 19 % and 25 % in Tanzania and between 19 % and 44 % in Uganda, consistent with other studies from sub-Saharan Africa [23–26]. The prevalences of other NCDs were comparatively low. Risk factors for NCDs were also common in both countries, although their prevalence varied between men and women, with smoking and alcohol consumption being more prevalent among men, while obesity and reported lack of physical activity occurred more frequently among women. Obesity was particularly common, exceeding 15 % among women in some strata. Age-standardized HIV prevalence was high and consistent with data from national statistics in both countries [27, 28].

Hypertension prevalence was higher in rural than urban areas, in contrast to a 2007 review [23], but in line with some more recent studies [29–31]. This is consistent with the high prevalence of some NCD risk factors in rural areas in our study, and suggests that life style and dietary changes are increasingly affecting rural areas in SSA. As expected, hypertension prevalence increased with age, exceeding 40 % in several strata among those ≥45 years old. However, hypertension was observed even at comparatively young ages, particularly among men, in agreement with some other studies in SSA [32]. Being overweight increased the odds of being hypertensive, as expected [33–35], with double the risk for individuals with central obesity. However, the proportion of cases of hypertension attributable to the joint effect of obesity measured by BMI and waist circumference was only 28 %; this contrasts with studies from Europe and the US where two-thirds of hypertensive cases were attributable to these factors [33, 36]. In addition to behavioral and anthropometric risk factors, other causes including salt sensitivity, subclinical renal disease, chronic inflammation and/or genetic factors may be playing a role in the pathogenesis of hypertension in our region [37–40]. Perhaps unexpectedly, current smoking was associated with a lower risk of hypertension; however, inconsistent or inverse associations between smoking and blood pressure have been described by others [41, 42]. It is possible that the effect is due to unrecorded or uncontrolled confounding factors.

DM was much less common than hypertension, with an overall observed prevalence of about 1 % in Tanzania and 3 % in Uganda. The prevalence in Tanzania is notably lower than the 9 % prevalence observed in the recent Tanzanian national STEPS survey [43]. This difference is at least partly attributable to the different age bands included (25 to 64 years compared to 18+ years as in our study) and due to the inclusion of individuals with pre-diabetes in the national survey (FBG ≥6.1 mmol/L compared to ≥7 mmol/L as in our study). Diabetes prevalence varies widely across SSA, from 1 % in rural Uganda to 12 % in urban Kenya [44]. The prevalence of heart failure varied, with highest levels in Entebbe town (9 %) and lowest in Tanzanian district towns (1 %). Hypertension is the main cause of heart failure in Africa, and it may be predicted that the prevalence and incidence of heart failure in Africa will rise due to the high burden of uncontrolled arterial hypertension [4, 45, 46].

We were not able to distinguish COPD from asthma in this study as it was not possible to apply a bronchodilator before measuring FEV1 and FCV as is recommended by the Global Initiative for Chronic Obstructive Lung Disease [17]. In our study, using an age cut-off of 30 years and assuming that most COPD occurs above this age, the prevalence of COPD would be between 1 % and 6 % in different strata, with higher levels in rural areas. Epilepsy was defined based on self-report, yielding a prevalence of 0 % to 2 %. This is likely to be an underestimate: qualitative studies conducted in study communities soon after our survey revealed that epilepsy is a highly stigmatized disease in both countries (Janet Seeley and Soori Nnko: personal communications), confirming observations from others [47, 48]. On the other hand, our findings were in line with those from a study using data from five demographic surveillance systems from SSA which reported prevalences among adults of 0.5 % to 1.5 % [49].

Lack of awareness was common among people with hypertension and other CDs, particularly in rural areas. Frequent lack of awareness and its negative impact on health outcomes has been documented in other studies and is a major barrier to control [32, 50]. In patients with hypertension, the prevalence of awareness was somewhat higher among ex-smokers, so it is possible that they may have quit smoking after diagnosis; but it was also higher among people who were inactive and overweight, suggesting that these groups may have been ill and therefore sought care, or alternatively that awareness may not necessarily translate into the adoption of a healthier lifestyle. Major efforts are needed to educate both the general public and individual patients about the threat these NCDs pose to health, and to promote a healthy lifestyle with regards to diet, physical activity and appropriate body weight control [44, 50, 51]. However, this is likely to be a protracted battle in societies that were historically mainly exposed to acute, infectious health problems, and among whom malnutrition was common and, consequently, overweight is still regarded as a sign of health and wealth [52, 53].

A major strength of our research is the relatively large sample size of our binational study and the carefully-conducted sampling procedure which ensured population representativeness for the areas studied. Another strength is the use of the STEPS questionnaire as a standardized interview tool recommended by WHO for studies on NCDs [15], which allows comparison between our study and others. Our results are strikingly similar across the two countries, in particular with respect to the high prevalence of hypertension and the risk factors known to be associated with hypertension and diabetes, thus emphasizing the urgency of our call for effective public health interventions. The survey is part of a larger research program in Uganda and Tanzania that focuses on selected NCDs and HIV infection, based on the rationale that, from a health services perspective, these conditions have a number of commonalities [54, 55].

Our study has some weaknesses: There are other important CDs such as cancers that were not covered by our survey. CDs were investigated using a variety of diagnostic procedures. For HIV, hypertension and diabetes, diagnoses were made using highly sensitive and specific tests. For obstructive lung disease, chronic heart failure and epilepsy, we were restricted by what was feasible in the field. The question about physical activities may have been misunderstood by some survey participants, particularly in Uganda where the proportion reporting vigorous activity was much lower than in Tanzania. However, data on lack of physical activity from Tanzania from our survey (39 % overall) were comparable to those from the national STEPS survey (32 %) [43]. The selection of clusters within 5 km of a health facility may have led to overestimation of the proportion of CD patients diagnosed and treated in rural areas, implying that the degree of under-treatment may have been even larger than reported. However, most rural communities have health facilities, and most people do not live far away from them.

Our findings have a number of implications for policy and practice in Tanzania and Uganda and probably elsewhere in SSA. The introduction of active case detection and intensive health education for hypertension and diabetes in the general population is urgently needed. Efforts are also needed to optimize linkage of NCD patients to care and ensure their regular follow-up [56]. Health services will need to be generally strengthened to meet the increasing demand, and we suggest that NCD services could learn from the successful introduction of HIV care to peripheral public health services in many African countries. Such efforts should focus on three major targets: (1) provision of clinical guidelines, basic diagnostic equipment, and first- line drug therapy for NCDs to all health centres and dispensaries; (2) strengthening of management systems for NCDs to provide regular training, supervision, and reporting; and (3) ensuring sufficient knowledge and experience related to NCDs among front-line health-care workers [55].

The high prevalence of NCD risk factors and the fact that the prevalence of diabetes is still comparatively low in our areas provide a window of opportunity and call for the urgent introduction of population level and inter-sectorial interventions to reduce population exposures to risk factors [57]. Educational mass media campaigns should be launched to increase awareness about the threat posed by NCDs and to promote a healthy life style. At schools, physical activity and traditional diets should be promoted and actively practiced to the extent possible. Existing legislation and taxation should be modified aiming to reduce the consumption of tobacco, alcohol and unhealthy food and beverages. Such inter-sectorial preventive measures are reflected in the emerging national NCD programs in Uganda and Tanzania, and are in line with recommendations from WHO and the United Nations [58, 59]. The effectiveness of such interventions has been demonstrated in industrialized countries [60–62].

## Conclusions

In this population-based survey of selected CDs from northwestern Tanzania and southern Uganda, hypertension prevalence was high and the majority of affected persons were not aware of their condition, let alone treated, representing a substantial unmet health need. Risk factors for hypertension and other NCDs were very common, suggesting that the burden of other NCDs, such as diabetes and heart failure, is set to increase. The fact that their prevalence is still substantially lower in East Africa than that of hypertension offers a window of opportunity for prevention efforts at the population level through educational campaigns and modified legislation and taxation, and at the individual level through risk factor screening and early disease detection. Such interventions must be implemented urgently. Health services need to be strengthened so that they will become able to cope with the increasing burden of newly-diagnosed NCD patients.

## Additional files

Additional file 1:
**Sampling methods – further details.**
Additional file 2:
**Supplementary Table - Factors associated with prevalence of untreated stage II hypertension among participants with hypertension in Tanzania and Uganda.**

## Abbreviations

aOR: adjusted odds ratio
ART: antiretroviral therapy
AUDIT: Alcohol Use Disorders Identification Test
BP: blood pressure
BMI: body mass index
CD: chronic disease
CI: confidence interval
COPD: chronic obstructive pulmonary diseases
DM: diabetes mellitus
FBG: fasting blood glucose
FEV1: forced expiratory volume in the first second
FVC: forced vital capacity
HH: households
HIV: human immunodeficiency virus
IQR: interquartile range
N: n, number
NCD: non-communicable disease
OR: odds ratio
PAF: population attributable fraction
PND: paroxysmal nocturnal dyspnea
RBG: random blood glucose
SSA: Subsaharan Africa
STEPS: STEPwise approach to surveillance of NCD risk factors (WHO)
USD: Unites States dollars
WHO: World Health Organisation
