# Supplementary material for: High prevalence of hypertension and of risk factors for non-communicable diseases (NCDs): a population based cross-sectional survey of NCDS and HIV infection in Northwestern Tanzania and Southern Uganda
Source: BMC Med. 2015 May 29;13:126. doi: 10.1186/s12916-015-0357-9 (PMC4476208; doi:10.1186/s12916-015-0357-9)
Supplement: Additional file 1: — Sampling methods – further details. [file 12916_2015_357_MOESM1_ESM.docx]

**Additional File 1. Sampling methods – further details**

As outlined in the main text, we used stratified, multistage sampling, with five strata in each country: a city/urban area (Mwanza city in Tanzania; Entebbe town in Uganda), two district towns (Geita and Kahama in Tanzania; Wakiso and Mpigi in Uganda) and two rural communities from the same districts (Figure 1). We took an independent two-stage self-weighting sample from each stratum.

Firstly, we randomly sampled nine clusters, defined as the lowest administrative local authority area (street/kitongoji in Tanzania; Local Council-1 (LC-1) in Uganda), with probability proportional to size (number of households). Because the survey was part of a larger research programme that ultimately aims to improve the existing health services for chronic diseases, and under the assumption that most health care seekers residing in district towns and rural areas within a 5 km radius of a health facility would use that facility, we restricted our sampling to clusters that are located within a 5 km radius of a larger health facility (hospitals or health centres in Tanzania; hospitals or health centres III or IV in Uganda) in these strata. Secondly, we randomly sampled households within clusters. Of note, Tanzanian “streets” in the urban stratum are larger entities and include more households than Ugandan LC-1s. In Uganda, the sample was self-weighting within strata; however, the probability of selection differed between the five sampling strata. In Tanzania, in addition to the differing selection probabilities between strata, households were selected using GPS coordinates, rather than council lists (see below), so the number of households per cluster varied slightly and the sample was only approximately self-weighting within strata. Therefore, sampling weights were applied to estimate the population prevalence of CDs and of potential NCD risk factors, within each country and location (municipalities, district towns, and rural areas).

The original sample size calculation assumed enrolment of ten households per cluster and an average of 1.76 adults per household (1), yielding a sample size of approximately 800 adults per country. The precision with which the chronic disease prevalences would be estimated depends on the prevalence and the "design effect" (2). For example, for a prevalence of 5%, we could estimate it in each of the countries with a precision of ±1.7% or ±3.3% with 95% confidence if the design effect is 1 or 3, respectively. For a prevalence of 10%, the corresponding figures are ±2.3% and ±4.3% (note that for small prevalences, the 95% confidence limits are not symmetric around the prevalence, and the precision applies to the upper confidence limit; the lower limit is obviously always >0). At the first Scientific Advisory Committee meeting on March 22, 2012, it was agreed to increase the number of households sampled from rural clusters to 15 in order to (a) obtain higher precision for the prevalence estimates of CDs in rural areas which are expected to be lower for some diseases, and (b) increase the representation of rural areas where the majority of the population lives. This decision increased the expected number of individuals to be enrolled in each country from 800 to 950 (Figure). The approved protocol permitted such adjustments.

In Uganda, lists of eligible local councils (LC-1s) and the number of households in each were obtained from the Uganda Bureau of Statistics, and households were selected from household lists available from the LC-1 authorities. The individuals enrolled for a given household were weighted as follows:

Let $n_{ij}$ be the number of households in stratum $i$ and cluster $j$ as per the council list, and $n_{i.}$ be the number of households in stratum $i$. Then the probability of a particular cluster $j$ in stratum $i$ being selected is approximately $p_{ij}=9*n_{ij}/n_{i.}$. Let $x$ be the fixed number of households selected in a given cluster (10 for urban clusters and 15 for rural clusters), and $m_{ij}$ be the actual number of households in stratum $i$ and cluster $j$ as per the household list obtained in the field for the chosen clusters. Then the probability of a particular household in given stratum $i$ and cluster $j$ being selected is $q_{ij}=x/m_{ij}$. Therefore the overall probability of a particular household being selected is $r=p_{ij}*q_{ij}=9*n_{ij}*x/(n_{i.}*m_{ij})$. In Uganda, it was assumed that the sampling frame was reasonably accurate, and therefore $n_{ij}=m_{ij}$, and so we simply have $r=9*x/n_{i.}$, and the sampling weight for that household is $1/r$.

In Tanzania, where household lists are typically not available, we mapped cluster boundaries using a global positioning system (GPS) device and randomly selected GPS coordinates (15 in Mwanza city and district towns, and 25 in rural areas) within a cluster using ArcGIS software version 9.3 (Esri, Redlands, USA). The GPS coordinates were randomly ordered and visited in that order, until the required number of households was identified. All households whose front doors were within 20 meters of each visited GPS coordinate were invited to enrol even if this would occasionally lead to exceeding the target number.

Using the same notation as above, we again have $p_{ij}=9*n_{ij}/n_{i.}$. However, the probability of a particular household in a given stratum $i$ and cluster $j$ being selected is now $q_{ij}=y_{ij}/n_{ij}$ where $y_{ij}$is the number of households identified at all of the GPS coordinates visited in cluster $j$ of stratum $i$. The overall probability of a particular household being selected is therefore $r=p_{ij}*q_{ij}=9*y_{ij}/n_{i.}$ and as above the weight is given by $1/r$.

In some rural areas in Tanzania with low density housing, 25 GPS coordinates were visited but only <15 households could be found. In this situation, it was possible to generate a household list with the help of local people for the random selection of households. The same methods as for Uganda were then applied for the sampling and weight estimation (without the assumption of $n_{ij}=m_{ij}$ since the sampling frame was not believed to be reliable).

A limitation of these methods is that we assume that the numbers of households per cluster and the household lists are accurate, although in practice it is likely that the council list will underestimate the number of households, due to the growing populations. This means that the probability of household selection may be overestimated and the weights are lower than they should be.

All selected households were visited and verbal consent from the household heads obtained before inviting all resident adults ≥18 years to participate. No replacements were made for households that refused participation or who could not be contacted after repeated visits.

**References**

1. Shafer LA, Maher D, Weiss HA, Levin J, Biraro S, Grosskurth H. Contribution of population factors to estimation of human immunodeficiency virus prevalence trends: a cohort study in rural Uganda, 1989-2007. Am J Epidemiol. 2011;174(10):1175–82.

2. Kish L. Survey sampling. New York: Wiley; 1965. p. 8.2.

**Figure 1. Study design.**

**Individual**

**Household**

**Cluster**

**Stratum**

Uganda

Tanzania

Mwanza

Geita town

Geita rural

Kahama urban

Kahama rural

Entebbe

Wakiso town

Wakiso rural

Mpigi urban

Mpigi rural

**Country**

Randomly select 9 clusters within each stratum, with probability proportional to size (no. clusters per country = 5*9 = 45)

Randomly select 10 households in each urban/district town cluster (no. households per country = 3*9*10 = 270)

and 15 households in each rural cluster (no. households per country = 2*9*15 = 270), therefore total 540 households

Within each household, aim to enrol all resident adults (≥18 years)

(no. individuals per country assuming average 1.76 adults/household = 540*1.76 = 950)
