# Supplementary material for: High prevalence of hypertension and of risk factors for non-communicable diseases (NCDs): a population based cross-sectional survey of NCDS and HIV infection in Northwestern Tanzania and Southern Uganda
Source: BMC Med. 2015 May 29;13:126. doi: 10.1186/s12916-015-0357-9 (PMC4476208; doi:10.1186/s12916-015-0357-9)
Supplement: Additional file 2: — Supplementary Table - Factors associated with prevalence of untreated stage II hypertension among participants with hypertension in Tanzania and Uganda. [file 12916_2015_357_MOESM2_ESM.docx]

**Additional File 2**

**Supplementary Table: Factors associated with prevalence of untreated stage II hypertension^1^ among participants with hypertension^2^ in Tanzania and Uganda**

|  | **n with stage II hypertension / total N (%)**^3^ | **Age-, sex- and stratum adjusted OR (95% CI) ^4^** | **Adjusted OR  (95% CI)** ^4^**^,5^** |
| --- | --- | --- | --- |
| **SOCIODEMOGRAPHIC** |  |  |  |
| **Age group** |  | **P < 0.001** |  |
| < 35 years | 17 / 98 (17.3%) | **1** |  |
| 35–49 years | 32 / 106 (30.2%) | **1.75 (0.99 -3.10 )** |  |
| ≥ 50 years | 81 / 164 (49.4%) | **4.64 (2.68 -8.02 )** |  |
| **Sex** |  | **P=0.004** |  |
| Male | 50 / 188 (26.6%) | **1** |  |
| Female | 80 / 180 (44.4%) | **2.05 (1.27 -3.32 )** |  |
| **Marital status** |  | P=0.82 |  |
| Married/living as married | 70 / 213 (32.9%) | 1 |  |
| Divorced/separated/widowed | 52 / 110 (47.3%) | 1.17 (0.71 -1.94 ) |  |
| Single | 8 / 45 (17.8%) | 0.87 (0.32 -2.35 ) |  |
| **Education** |  | P=0.29 |  |
| Secondary or above | 20 / 80 (25.0%) | 1 |  |
| Primary | 40 / 128 (31.3%) | 1.27 (0.61 -2.64 ) |  |
| None/incomplete primary | 70 / 160 (43.8%) | 1.69 (0.87 -3.31 ) |  |
| **Monthly income (USD)** |  | P=0.93 |  |
| Top tertile | 30 / 102 (29.4%) | 1 |  |
| Middle tertile | 40 / 114 (35.1%) | 1.03 (0.52 -2.01 ) |  |
| Lower tertile | 60 / 152 (39.5%) | 1.12 (0.56 -2.27 ) |  |
| **BEHAVIOURAL** |  |  |  |
| **Smoking** |  | P=0.75 |  |
| Never smoked | 107 / 296 (36.1%) | 1 |  |
| Ex–smoker | 10 / 34 (29.4%) | 0.72 (0.28 -1.82 ) |  |
| Current smoker | 13 / 38 (34.2%) | 0.83 (0.28 -2.41 ) |  |
| **Alcohol consumption** |  | P=0.41 |  |
| Never drinks/no drinking in past 12m | 87 / 254 (34.3%) | 1 |  |
| Non-problem drinking^6^ | 32 / 90 (35.6%) | 1.04 (0.62 -1.76 ) |  |
| Problem drinking^6^ | 10 / 23 (43.5%) | 1.79 (0.76 -4.20 ) |  |
| **Eats fruit/veg fewer than 5 days/week** |  | P=0.78 |  |
| No | 76 / 220 (34.5%) | 1 |  |
| Yes | 54 / 148 (36.5%) | 1.10 (0.56 -2.15 ) |  |
| **Days of vigorous physical activity/week** |  | P = 0.53 |  |
| None | 90 / 232 (38.8%) | 0.97 (0.53 -1.76 ) |  |
| 1–4 | 6 / 30 (20.0%) | 0.53 (0.17 -1.63 ) |  |
| 5+ | 34 / 106 (32.1%) | 1 |  |
| **ANTHROPOMETRIC** |  |  |  |
| **BMI category (kg/m^2^)** |  | P = 0.75 | P = 0.53 |
| Underweight (<18.5) | 13 / 29 (44.8%) | 1.42 (0.46 -4.38 ) | 1.58 (0.53 -4.74 ) |
| Normal (18.5–<25) | 65 / 209 (31.1%) | 1 | 1 |
| Overweight (25–<30) | 29 / 82 (35.4%) | 0.98 (0.54 -1.79 ) | 0.63 (0.30 -1.33 ) |
| Obese (≥30) | 19 / 41 (46.3%) | 1.45 (0.63 -3.35 ) | 0.87 (0.33 -2.29 ) |
| **Waist circum >94 cm (males)/>80cm (females)** |  | P=0.10 | **P=0.10** |
| No | 67 / 229 (29.3%) | 1 | **1** |
| Yes | 62 / 137 (45.3%) | 1.57 (0.92 -2.69 ) | **1.57 (0.92 -2.69 )** |

^1^Systolic BP ≥160/diastolic BP ≥100 in 3rd measurement at a single visit. ^2^Systolic BP ≥140 and/or diastolic BP ≥90, in 3rd measurement at a single visit. Excludes those on treatment for hypertension (N=9 in Tanzania and N=21 in Uganda). ^3^Actual number of respondents and proportion with stage II hypertension, without sampling weights applied. ^4^Standard errors adjusted for clustering in survey design. ^5^Sociodemographic and behavioural factors adjusted for age, sex, stratum ( a priori); there were no additional independent sociodemographic or behavioural predictors of stage II hypertension, so adjusted ORs are the same as in Column 2. Anthropometric factors adjusted for age, sex, stratum, and all independent anthropometric predictors of stage II hypertension: waist circumference category. ^6^Based on AUDIT screening tool. Non-problem drinking defined as AUDIT score <8; problem drinking defined as AUDIT score ≥8.
